# Apology and forgiveness evolve to resolve failures in cooperative agreements

**DOI:** 10.1038/srep10639

**Published:** 2015-06-09

**Authors:** Luis A. Martinez-Vaquero, The Anh Han, Luís Moniz Pereira, Tom Lenaerts

**Affiliations:** 1 AI lab, Computer Science Department, Vrije Universiteit Brussel, Pleinlaan 2, Brussels, 1050 Belgium; 2 MLG, Département d’Informatique, Université Libre de Bruxelles, Boulevard du Triomphe CP212, Brussels, 1050 Belgium; 3 School of Computing, Teesside University, Borough Road, Middlesbrough, TS1 3BA, UK; 4 NOVA Laboratory for Computer Science and Informatics, Departamento de Informática, Faculdade de Ciências e Tecnologia, Universidade Nova de Lisboa, Caparica, 2829-516 Portugal

## Abstract

Making agreements on how to behave has been shown to be an evolutionarily viable strategy in one-shot social dilemmas. However, in many situations agreements aim to establish long-term mutually beneficial interactions. Our analytical and numerical results reveal for the first time under which conditions revenge, apology and forgiveness can evolve and deal with mistakes within ongoing agreements in the context of the Iterated Prisoners Dilemma. We show that, when the agreement fails, participants prefer to take revenge by defecting in the subsisting encounters. Incorporating costly apology and forgiveness reveals that, even when mistakes are frequent, there exists a sincerity threshold for which mistakes will not lead to the destruction of the agreement, inducing even higher levels of cooperation. In short, even when to err is human, revenge, apology and forgiveness are evolutionarily viable strategies which play an important role in inducing cooperation in repeated dilemmas.

Recently, our innate capacity to create, and commit to, prior agreements^1^,^2^,^3^ has been proposed as an evolutionarily viable strategy inducing cooperative behavior in social dilemmas. It provides an alternative to different forms of punishment of inappropriate behavior, or of rewards to stimulate the proper one^4^,^5^,^6^,^7^,^8^. Commitments – defined as prior agreements with potentially posterior compensations in case the agreements fail – are wide-spread in human societies at different scales, from personal relationships such as marriage to international and organisational ones such as alliances among companies and countries^1^,^2^,^3^,^9^,^10^. Anthropological data reveals that commitment strategies, as for instance demand-sharing^11^, have played an essential role in early hunter-gatherer societies. A recent body of economic experiments show that arranging prior commitments promote cooperation in diverse scenarios from one-shot to repeated games^12^,^13^,^14^. Analytical and numerical methods have shown that commitments are evolutionarily viable when the cost of arranging them is sufficiently small compared to the cost of cooperation both in the one-shot pairwise prisoners dilemma^15^ and the one-shot public goods game^16^.

However, commitment deals, like some examples mentioned earlier, are most often established to ensure favourable interactions over longer time periods, implying repeated encounters between the actors that established the agreement, as well as the appeal of repeated benefits. Experiments have shown that commitment facilitates cooperation in long-term interactions^13^,^17^, especially when it is voluntary. Moreover, long-term commitments are most likely more cost-efficient as the cost of setting up the agreement is paid only once for the entire duration of the agreement. Interestingly, commitment may also induce behavioral differences in repeated games: As suggested in^2^, the individuals’ preferred behavior in repeated interactions may shift from a conditional reciprocal to an unconditionally cooperative behavior, which will indeed be confirmed analytically and numerically in this manuscript.

Using methods from Evolutionary Game Theory^18^,^19^, we provide for the first time analytical and numerical insight into the viability of commitment strategies in repeated social interactions, which will be modeled through the Iterated Prisoners Dilemma (IPD)^20^. In order to study commitment strategies in the IPD a number of behavioral complexities need to be addressed. First, agreements may end before the recurring interactions are finished. As such, strategies need to take into account how to behave when the agreement is present and when it is absent, on top of proposing, accepting or rejecting such agreements in the first place. Second, as it was shown within the context of direct reciprocity^21^, individuals need to deal with mistakes made by the opponent or by themselves, caused for instance by “trembling hands” or “fuzzy minds”^19^,^22^: A decision needs to be made on whether to continue the agreement, or end it collecting the compensation resulting from the other’s defection.

As errors might lead to misunderstandings or even breaking of commitments, individuals may have acquired sophisticated strategies to ensure that mistakes are not repeated or that profitable relationships may continue. Revenge and forgiveness may have evolved exactly to cope with those situations^23^,^24^: The threat of revenge, through some punishment of withholding of a benefit, may discourage interpersonal harm. Yet often one cannot distinguish with enough certainty if the other’s behavior is intentional or just accidental^25^,^26^. In the latter case, forgiveness provides a restorative mechanism that ensures that beneficial relationships can still continue, notwithstanding the initial harm. An essential ingredient for forgiveness, analysed in this work, seems to be (costly) apology^23^, a point emphasised in^27^.

The importance of apology and forgiveness for sustaining long term relationships has been shown in different experiments^28^,^29^,^30^,^31^. Apology and forgiveness are of interest as they remove the interference of external institutions, which can be quite costly to all parties involved, in order to ensure cooperation. Evidence shows that there is a much higher chance that customers stay with a company (they hence forgive) that apologises for mistakes^28^. Apology leads to fewer lawsuits with lower settlements in medical error situations^32^. Apology even enters the law as an effective mechanism of resolving conflicts^33^,^34^. Hence, it is important to know how apology and forgiveness can help coping with misunderstanding, on either side, in an internal way, without jeopardising the ongoing commitment. Even without explicit apology, the participants in an IPD seem to use a form of implicit apology, by cooperating in several subsequent rounds after making a mistake^35^,^36^,^37^. Yet, such an unclear apology might not thoroughly resolve the misunderstanding, as is the case for TFT-like strategies^35^,^36^,^38^,^39^].

In this work, once the viability of the commitment strategy within the context of the IPD is analysed, we analytically and numerically determine when explicit apology and forgiveness are evolutionarily viable, and how sincere apology needs to be for forgiveness, thus sustaining the mutually beneficial relationship, which so far we are aware have never been provided.

## Results

### Defining all strategies

We consider a finite population of *N* individuals, with *N* = 100 in our analysis. At the beginning of each generation, individuals are randomly matched to play an IPD game. In each round of this game they can either cooperate (C) or defect (D), acquiring a payoff given by the Donation game^19^ — an instance of the PD – as represented by the following parametrised payoff matrix with *b *> *c*:


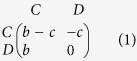


With a probability *ω* the encounter between two individuals is repeated for another round, leading to an average number of rounds per IPD interaction *R*_*T*_ = (1–*ω*)^−1^. Individuals may make implementation mistakes, *i.e*. playing D when they intend to play C and vice versa, with a probability *α*. At the end of a generation, more successful individuals – those that accumulated higher total payoffs – are more likely to be imitated by less successful individuals (see Methods for more details).

At the first encounter before playing the IPD, individuals can agree to play C in every round of the game. To set up a commitment one of the players has to propose it, at a cost *ε* (players share that cost if both are proposers), while the co-player needs to decide whether to accept it. The commitment lasts as long as both players fulfill their commitment, *i.e*. they play C. If one defects then she has to pay a compensation *δ* to the other player and the commitment is broken.

When neither player proposes a commitment or the commitment ends, both individuals play a reactive strategy^35^, which is modelled by a triplet (*p*_0_,*p*_*C*_,*p*_*D*_): *p*_0_ represents the probability of cooperating in the first round, *p*_*C*_ the probability of cooperating in the current round if in the previous round the co-player cooperated and similarly for *p*_*D*_, which is the probability of cooperating in the current round if the co-player defected in the previous round. In this work, we will only consider pure reactive strategies in the presence of noise (*α*): always cooperating AllC, (1 – *α*,1 – *α*,1 – *α*), always defecting AllD (*α*,*α*,*α*), TFT (1 – *α*,1 – *α*,*α*) and *anti*-TFT (ATFT) (1 – *α*,*α*,1 – *α*). All these strategies, except AllD, play C in the first round.

Thus, the full strategy *S*_*i*_ of any individual in our model is defined by three parameters *S*_*i*_ = (*S*_*c*_,*S*_*in*_,*S*_*out*_)_*i*_, where

- *S*_*c*_ ∈ {*P*,*A*,*NC*} represents whether the strategy is a *proposing* player who proposes and accepts commitments (P), an *accepting* player who does not propose a commitment but accepts those that are proposed (A), or a *non-committing* player that never accepts a commitment proposal strategy (NC). We consider at this point only simultaneous interactions, meaning that when both players propose to commit they do this at the same time, hence sharing the commitment cost.
- *S*_*in*_ ∈ {*C*,*D*} indicates the behavior the player chooses when she is in a commitment: cooperating with a probability 1–*α* (C) or *α* (D). Non-committers do not have any *S*_*in*_ strategy since they never participate in commitments, which will be represented by *S*_*in*_ = “–’’ in the strategy *S*_*i*_.
- *S*_*out*_ ∈ {*AllC*,*AllD*,*TFT*,*ATFT*} represents the reactive strategy chosen by the player when not in a commitment.

Hence the strategy of a defector or a cooperator is represented here by, respectively, (*NC*,–,*AllD*) and (*A*,*C*,*AllC*). A strategy that proposes a commitment, and honours the agreement, while defecting when the agreement is broken is represented by (*P*,*C*,*AllD*). The strategies FAKE and FREE, discussed in^15^, could be represented by (*A*,*D*,*), with “*’’ representing any of the *S*_*out*_ options, and (*A*,*C*,*) respectively.

Finally, we need to consider that when the commitment is broken, or when the agreement is not created, individuals can decide to simply stop the game, which results in a zero payoff for all following rounds. Proposers that do not play when the commitment is broken will not have an *S*_*out*_ strategy. These two possibilities lead to four different scenarios:

- **PP**: individuals continue to play their reactive strategies both when the commitment is not set up and after an established commitment is broken,
- **NP**: they play their reactive strategies once a commitment ends, but stop interacting if a commitment is rejected (even if additional rounds could be played),
- **PN**: individuals play their reactive strategy if a proposed commitment is not accepted, but stop interacting if a commitment is broken,
- **NN**: individuals refuse to play in any of these situations.

Given these four scenarios, one can identify 20 strategies for PP, NP and PN scenarios, and 14 strategies for the NN scenario. The number of strategies will increase when an apology-forgiveness mechanism is introduced, as is shown later. It is not clear which of these scenarios leads to the most cooperative outcome. Our results, as discussed below, aim to reveal, when individuals can choose by themselves which scenario to use, which of the four scenarios is evolutionary more viable than the other. In other words, is it best to always play the game, using their *S*_*i*_ strategies or should one refuse the interaction when the agreement cannot be established or when it is broken (or both)? The details on how the payoffs for each strategy are calculated are provided in Methods.

### The emergence of revenge after the commitment is broken

We first study commitments in the absence of an apology-forgiveness mechanism. In this situation, there are two main differences with the model in^15^ next to the iterated nature of the game: the inclusion of noisy C and D actions and how players react when commitments fail or cannot be established (previously called *scenarios PP, PN, NP* and *NN*).

**Figure 1:**
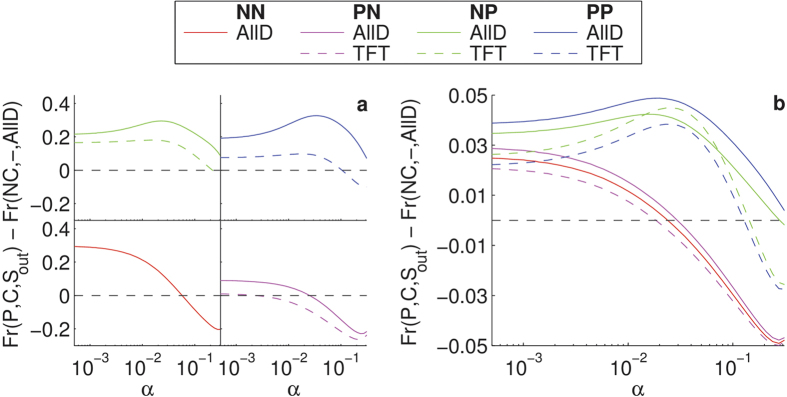
Success of commitments and revenge after commitments break. Stationary distribution of the most dominant strategies (proposers that cooperate within the commitment) relative to the stationary distribution of the pure defectors as a function of noise for PP, NP, PN and NN scenarios separately (**a**) and together (**b**). Different lines correspond to different *S*_*out*_. We assumed *ω* = 0.9, *b*/*c* = 2, *ε* = 0.25, and *δ* = 4.

In Fig. 1a, where the four scenarios are analysed separately, we plot for each scenario the frequencies of the most dominant strategies relative to the frequency of defectors (*i.e*. (*NC*,–,*AllD*) which do not commit when requested, hence have no *S*_*in*_, and always defect when no agreement is established or an established one is broken) as a function of the errors (*α*) that individuals can make while playing. These relative frequencies reveal for which noise levels the latter can suppress the defection strategy (see Supplementary Table 1 for the outcome for all strategies). In all four scenarios the dominating strategies are, for most noise levels, of the type (*P*,*C*,*), which correspond to proposers that cooperate as long as the commitment lasts and then behave reactively when the commitment is broken (see Supplementary Information for additional data on the results obtained for different conditions, including the benefit-to-cost ratio, the cost of establishing a commitment, and the penalty for breaking the commitment). One can see that in general the relative frequency of these proposers decreases as the noise level increases: The higher the noise the more likely a commitment is broken unintentionally. The scenarios PP and NP differ from the other two inasmuch as proposers can only sustain themselves in the latter for lower noise levels (*α* < 10^−1^). The (*P*,*C*,*AllD*) strategies are better off in all four scenarios, dominating in the PP and NP scenarios even for high noise levels and low benefit-to-cost ratios, which correspond to more severe social dilemmas. As in the PN and NN scenarios proposers can only sustain themselves for lower noise levels, commitment proposing strategies in IPD seem to be more successful if they are capable to actively take revenge by withholding the benefit (through defection) against individuals breaking commitments before the end of the game.

Commitment proposing strategies survive in combination with different types of accepting strategies (Supplementary Table 1). These accepting strategies are reminiscent of the FREE, *i.e*. the accepting strategies that cooperate when an agreement is established, and FAKE, *i.e*. the accepting strategies that defect in the commitment, types analysed before in the context of the one-shot PD and public goods games^15^,^16^. Interestingly, revenge also plays an important role here as, under highly erroneous conditions, these accepting strategies will also prefer to withhold the benefits from the proposer when the agreement ends. Note that the same transition from TFT to AllD, for higher noise levels, can be observed when no commitments are possible in the IPD (see Supplementary Figure 2).

Figure 1b confirms the earlier observation that proposing strategies are more successful when they can actively take revenge: if players can choose how to act outside of the commitment instead of having it imposed externally (in other words, each individual decides which one of the four scenarios to use in its strategy), the best strategies are those that defect (or play TFT in a few cases) after the commitment is broken. Hence, not playing when there is no agreement or when it is broken (NN) or only when the agreement is broken (PN) is less viable as a strategy than continuing to play in all situations (PP) or only refusing to play when no agreement can be established (NP). Interestingly, the possibility of proposing prior commitments changes the nature of the repeated game as it induces the emergence of revenge or retaliation rather than reciprocity or avoiding to interact (corresponding to the PN and NN scenarios) once commitment is broken^2^,^14^. This result is in contradiction to what happens when one does not have the option of proposing commitments in the IPD, where TFT is the most important strategy for *low levels of noise* (Supplementary Figure 2). As such, commitments reduce the advantage of TFT in comparison to AllD, altering the game and resulting in the situation where AllD becomes more viable than TFT.

**Figure 2:**
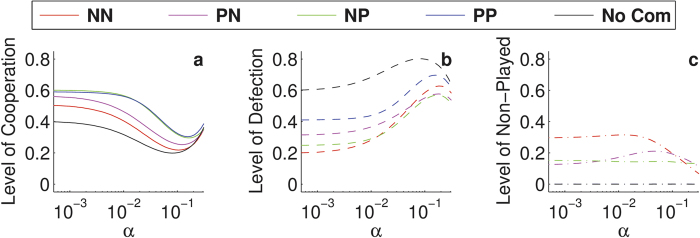
Commitments increase the level of cooperation. Levels of cooperation (**a**), defection (**b**) and non-playing (**c**) for the dominant strategies (proposers that cooperate within the commitment), as a function of the noise for the different scenarios. The black lines correspond to the situation where commitments cannot be made, serving as a baseline for the other approaches. We assumed *ω* = 0.9, *b*/*c* = 2, *ε* = 0.25, and *δ* = 4.

One could hypothesise that revenge may lead to a lower level of cooperation since proposers end up defecting when they are not in a commitment. Nevertheless, Fig. 2 reveals that the presence of these retaliating commitment proposing strategies (in each of the four scenarios) increases the level of cooperation: When comparing the black line to the coloured lines in that figure, cooperation increases (Fig. 2a) and defection decreases (Fig. 2b), yet this decrease hides that certain scenarios suffer from an increase in games not being played (Fig. 2c). For instance, although the level of defection in NN seems lower than in PP, one needs to take into account that not playing could be considered an alternative form of defection. Hence, when combining defection and not playing, the PP scenario has the highest level of cooperation (≈0.6) and the lowest level of defection (≈0.4), making it the best approach to induce cooperation in a population (see also Fig. 1b).

### Forgiveness requires a sincere apology to ensure cooperation

Introducing apology and forgiveness requires us to extend the strategy *S*_*i*_ with at least one additional parameter representing apology and forgiveness (for a more elaborate model description see Methods). Under the assumption that forgiveness occurs if and only if an apology took place, the apology parameter *q*_*apo*_ determines whether a player apologises after defecting, paying a compensation amount *γ* to the other player. With this definition, the strategy of a player *i* is now extended to *S*_*i*_ = (*S*_*c*_,*S*_*in*_,*S*_*out*_,*q*_*apo*_)_*i*_. We assume a strategy apologises when *q*_*apo*_ = 1 and does not when *q*_*apo*_ = 0.

We focus here on costly apology (γ > 0) and how it induces forgiveness as costless apology (γ = 0), being equivalent to forgiveness without apology, does not substantially change the conditions under which proposers are better than pure defectors: Forgivers only do better when the benefit-to-cost ratio is high enough (see Supplementary Figures 6 and 7).

**Figure 3:**
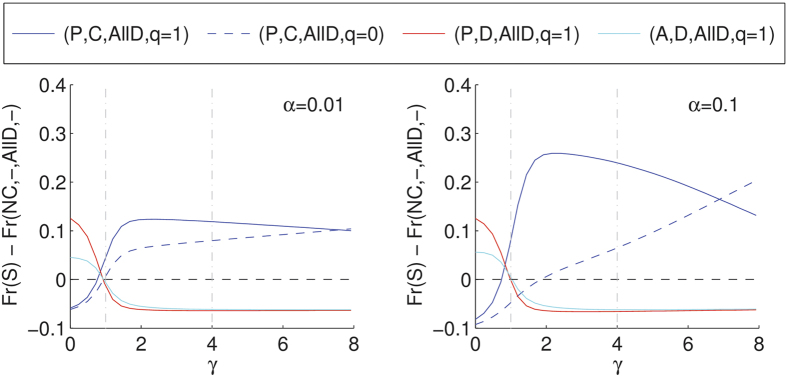
Forgiveness is evolutionary viable if apology is sincere. Stationary distribution of the main strategies with respect to the stationary distribution of the pure defectors as a function of the apology cost for the PP scenario and *α* = 0.01 (left) and *α* = 0.1 (right). Vertical dashed lines mark the values of *c* and *δ*. We assumed *ω* = 0.9, *b*/*c* = 2 (with *c* = 1), *ε* = 0.25, and *δ* = 4.

Figure 3 shows that when the compensation (γ) given upon apology is bigger than or equal to the cost of cooperating (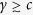), proposers that cooperate during commitments, apologise when they defect by mistake and forgive when receiving an equivalent apology become the best strategists in the IPD, in all scenarios (see also Supplementary Information). They reach a maximum when *c* < γ < *δ* and continue dominating the population until γ becomes too high (γ ≈ 7 for the PP scenario and 0.1 noise, and even higher for other scenarios, but with similar patterns; see Supplementary Information), leading to the situation where revenge, *i.e*. (*P*,*C*,*AllD*,*q*_*apo*_ = 0), becomes once again the better choice. However, when the cost of apology is not high enough (lower than *c*), fake proposers and acceptors, *i.e*. (*P*,*D*,*AllD*,*q*_*apo*_ = 1) and (*A*,*D*,*AllD*,*q*_*apo*_ = 1), take over. These fake proposers and acceptors systematically exploit the apology-forgiveness mechanism, leading to the decrease of cooperation. Hence our results show that apology needs to be sufficiently sincere, meaning not too low, not too high (δ > γ > c), in order for forgiveness to function properly, which intuitively makes a lot of sense. Actually, one can show that the cooperative proposer is a dominant strategy against the defecting proposer when *γ* > *c* and against the defecting acceptor when *γ* > *c* + 3*ε*/4 in the absence of noise and if all of them apologise (see Methods). According to Fig. 3, reducing the noise affects the importance of the apologising strategy (*P*,*C*,*AllD*,*q*_*apo*_ = 1) relative to the defecting and non-apologising strategy (*P*,*C*,*AllD*,*q*_*apo*_ = 0), yet the patterns described above remain valid.

**Figure 4:**
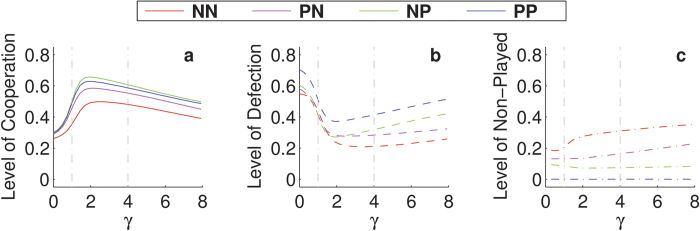
Sincere apology increases the level of cooperation. Levels of cooperation (**a**), defection (**b**) and non-playing (**c**) for the main strategies (proposers that cooperate within the commitment) as a function of the apology cost for the different scenarios. Vertical dashed lines mark the values of *c* and *δ*. We assumed *ω* = 0.9, *b*/*c* = 2, *α* = 0.1, *ε* = 0.25, and *δ* = 4.

Under the assumption of high noise levels (*α* ≈ 0.1), which reduces the level of cooperation (see Fig. 2), we can now see in Fig. 4 that apology plus forgiveness seriously boost the level of cooperation and reduce defection when *γ* > *c*. Yet if the apology is not sincere enough (*γ* < *c*) one can observe the opposite behavior, even in the PP scenario case. Introducing noise in the apology and forgiveness decisions does not generate qualitative differences in these conclusions. In Supplementary Information we analyse the influence of the average number or rounds (see Supplementary Figures 9-11), showing that in repeated interactions commitments become increasingly beneficial especially when after commitment is broken one takes apology and revenge into account.

### Evolution selects sincerity in apology and forgiveness

Clearly, the decision of how strongly to apologise or when to accept an apology are personal choices. As such, individuals can apologise at different costs γ and can forgive defection conditional on a personal threshold *τ*_γ_. Therefore, if the strategy is forgiving, the parameter *τ*_γ_ is used to decide whether the player will forgive the opponent or not.

Limiting here the analysis only to strategies that defect when they are not committing we determine which threshold and apology values evolve under natural selection. Reducing the number of strategies to these ones does not reduce the generality of the results, since they are the dominant ones (that always accumulate in almost 100% fraction of the population) as we have shown before.

**Figure 5:**
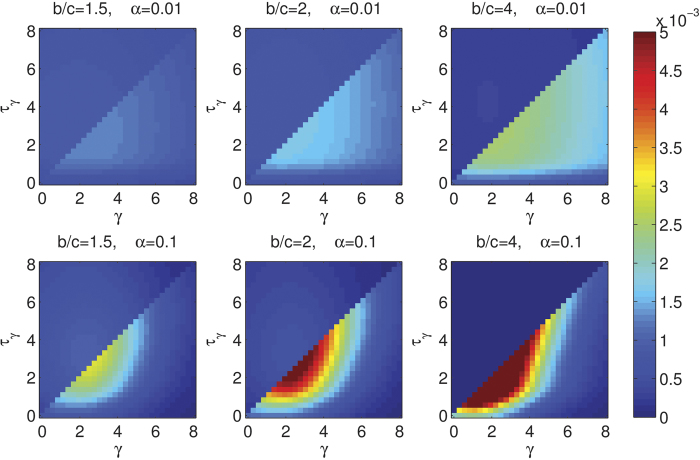
Thresholds for conditional forgiveness. Stationary distribution of cooperative proposers as a function of the cost of their apologies *γ* and the threshold *τ*_*γ*_ they require to forgive a co-player for the PP scenario. We assumed *ω* = 0.9, *ε* = 0.25 and *δ* = 4.

Figure 5 reveals which thresholds (*τ*_γ_) are preferred and which apologies (γ) are required for the PP scenario (additional results show almost the same results are obtained for the other scenarios). First one can observe that expecting a higher apology than one actually offered (*τ*_γ_ > γ) is always a bad strategy: in all the situations visualised in the figure, this situation leads to loss of cooperative commitment proposers, and hence cooperation in general. As was learned too from the results in Fig. 3 and Fig. 4, it is still not a good strategy to pay too high cost to apologise, as this behavior tends to disappear from the population. We see that the dominating strategies have apology values (γ) in the same region as the ones shown in Fig. 3. In Fig. 5 one can also observe that the higher the noise the more strategies converge to concrete values of γ and *τ*_γ_, in other words, the more important is the apology-forgiveness mechanism due to a higher number of mistakes, as also shown in Fig. 3. Yet, one can also observe in Fig. 5 that apology and forgiveness are less important in more severe games (*i.e*. very low benefit-to-cost ratios).

Nevertheless, results show that even in the case of individual choices, apology and forgiveness provide an important mechanism ensuring that commitments can remain stable and both parties can continue to profit from their original agreement.

## Discussion

Creating agreements and asking others to commit to such agreements provides a basic behavioral mechanism that is present at all the levels of society, playing a key role in social interactions^2^,^10^,^14^. Although it was shown that this behavior is evolutionary viable, little analytical and numerical insight is available on how to handle agreements and commitments in repeated interactions. The results discussed in this work fill this gap by clarifying and extending the observations made in experiments like^12^,^13^, while also showing that, similar to the one-shot interaction scenario, the introduction of ongoing subsisting commitments leads to higher levels of cooperation whenever the cost is sufficiently small and the compensation is high enough. Our work reveals how, when moving to repeated games, the detrimental effect of having a large arrangement cost is moderated as a subsisting commitment can play its role for several interactions. In these scenarios, the most successful individuals are those that propose commitments (and are willing to pay their cost) and, following the agreement, cooperate unless a mistake occurs. But if the commitment is broken then these individuals take revenge and defect in the remaining interactions, confirming analytically what has been argued in^23^,^24^. This result is intriguing as revenge by with holding the benefit from the transgressor may lead to a more favorable outcome for cooperative behavior in the IPD as opposed to the well-known reciprocal behavior such as TFT-like strategies.

Yet, as mistakes during any (long-term) relationship are practically inevitable, individuals need to decide whether it is worthwhile to end the agreement and collect the compensation when a mistake is made or whether it is better to forgive the co-player and continue the mutually beneficial agreement. To study this question the commitment model was extended with an apology-forgiveness mechanism, where apology was defined either as an external or individual parameter in the model. In both cases, we have shown that forgiveness is effective if it takes place after receiving an apology from the co-players. However, to play a promoting role for cooperation, apology needs to be sincere, in other words, the amount offered in the apology has to be high enough (yet not too high), which is also corroborated by a recent experimental psychology paper^40^. This extension to the commitment model produces even higher cooperation levels than in the revenge-based outcome. In the opposite case, fake committers that propose or accept to commit with the intention to take advantage of the system (defecting and apologising continuously) will dominate the population. In this situation, the introduction of the apology-forgiveness mechanism destroys the increase of the cooperation level that commitments by themselves produce. Hence there is a lower-limit on how sincere apology needs be as below this limit apology and forgiveness even reduce the level of cooperation one could expect from simply taking revenge. It has been shown in previous works that mistakes can even induce the outbreak of cheating or intolerant behavior in society^41^,^42^, and only a strict ethics can prevent them^42^, which in our case would be understood as forgiving just when apology is sincere.

Commitments in repeated interaction settings may take the form of loyalty^17^,^43^, which is different from our commitments regarding posterior compensations, which do not assume a partner choice mechanism. Loyalty commitment is based on the idea that individuals tend to stay with or select partners based on the length of their prior interactions. We go beyond these works by showing that, even without partner choice, commitment can foster cooperation and long-term relationships especially when accompanied with a sincere apology and forgiveness whenever mistakes are made.

A substantial body of economic experiments on commitments, apology and forgiveness, have been carried out, and the results from this work are in close accordance with the outcomes of those experiments^14^,^26^,^29^,^31^. In^14^, a PGG experiment shows that when commitment is arranged in advance, and set up afterwards, high levels of cooperation are observed. But if the commitment fails to form (i.e. some participants do not agree to commit), the players act significantly less cooperative than when they had no opportunity to join a commitment. This outcome is similar to the emergence of AllD strategy whenever commitment is not formed or when it is formed but then broken in our system. Next, several economic experiments show that apology only promotes cooperation when it is sincere, *i.e*. costly enough^26^,^29^,^31^. Ohtsubo’s experiment^31^ shows that a costlier apology is better at communicating sincerity, and as a consequence will be more often forgiven. This observation is shown to be valid across cultures^29^. In another laboratory experiment^26^, the authors showed apologies work because they can help reveal the intention behind the wrongdoers preceding offence. In compliance with this observation, in our model, an apology is mostly made by those who intended to cooperate but defect by mistake.

In conclusion, our results demonstrate that even when “to err is human”^44^, behaviors like revenge and forgiveness can evolve to cope with mistakes, even when they occur at high rates. On the other hand, mistakes are not necessarily intentional and even when they are it might still be worthwhile to continue a mutually beneficial agreement. Yet, as shown in this work, a sincerity threshold exists where the cost of apologising should exceed that of cooperation to induce the latter.

## Methods

### Payoffs under commitments

Payoffs introduced in the manuscript depend on the concrete strategies that players *i* and *j* decide to choose. A commitment is set up only if both players are proposers and as such, both share the cost of establishing it (
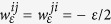
), or only one of the players (*i*) is a proposer and the other is an acceptor (*j*) and then only the first one has to pay that cost (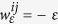 and 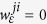). Denote 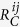 the number of rounds the players are, on average, in the commitment. Hence, 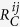 is a function of the probability that the commitment is not broken in the next round, denoted by Ω_*ij*_, and the probability that the IPD game continues for another round *ω*, which can be written as follows:


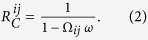


We denote by ***p***_*α*,*ij*_ the vector that represents the probability that players *i* and *j* actually play CC, CD, DC, and DD, respectively, in a round. The probability that the commitment continues once both players choose their actions depends on the apology-forgiveness mechanism and is represented by the vector ***q***_*c*,*ij*_ = (1,*q*_*ij*_,*q*_*ji*_,*q*_*ij*_*q*_*ji*_). Then


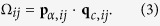


During the commitment, the *i*-player obtains a payoff per round.


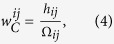



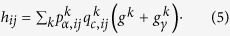


except in the last round, where she receives 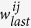. We have represented ***g*** = (*b* − *c*, −*c*, *b*, 0) as the vector that contains the payoffs coming directly from the IPD payoff matrix that the first player obtain in states (CC, CD, DC, DD). The vector ***g***_γ_ = (0, γ, −γ, 0) stands for the payoffs linked to the apologies needed to maintain the commitment when any player defects. The payoff received in the last round can be computed as


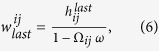



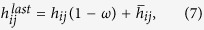


where 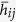 denotes the payoff that the *i*-strategist obtains if the commitment is broken:


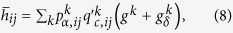



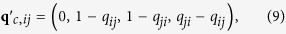



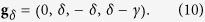


Note that the last element of ***q***'_*c*,*ij*_ and ***g***_*δ*_ vectors takes into account whether one or only one of the players forgives a mutual defective behaviour in the commitment.

Vector ***p***_*α*,*ij*_ depends on the strategies 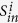 and 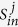, as well as on the noise, so that functions Ω_*ij*_ and *h*_*ij*_, and payoff 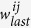 depend on them as well. Four different scenarios can be described as a function of these strategies:

- Both players intend to cooperate when they commit 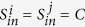:

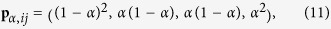



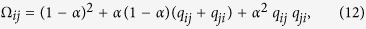



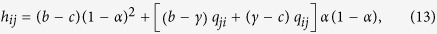



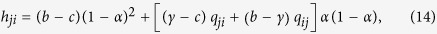



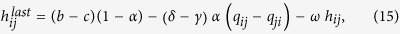



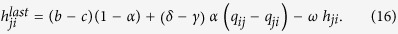

- Both players intend to defect in a commitment 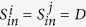:

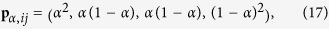



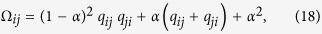



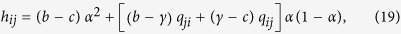



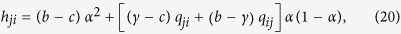



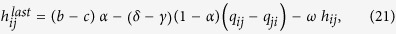



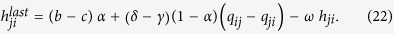

- Player *i* intends to cooperate and her co-player *j* intends to defect 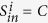 and 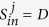:

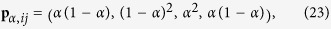



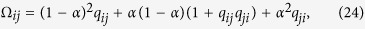



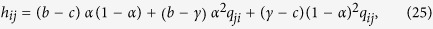



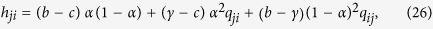



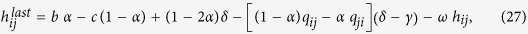



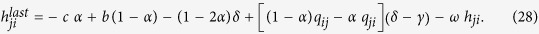

- Player *i* intends to defect and player *j* intends to cooperate 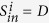 and 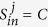. This case is equivalent to switch *i* and *j* indices in the previous case.

Since commitments last as far as nobody defects, 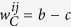 in the absence of any apology-forgiveness mechanism.

### Payoffs without commitments

When individuals play their reactive strategies *S*_*out*_, payoffs can be computed using the method described by^19^. In each round of this game there are four possible states (CC, CD, DC, DD) depending on the actions of player *i* and *j*. Taking into account that the action of a player in the current round is given by the action of the co-player in the previous one, the process can be described as a Markov chain in the state space. The stochastic matrix ***Q*** that represents the transition probabilities is given by


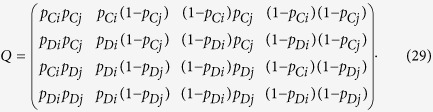


The initial probabilities for the four states are given by the vector


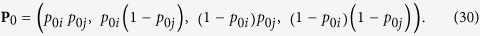


Then the total payoff that a *i*-strategist obtains playing with a *j*-strategist in the lack of commitments is


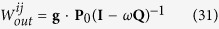


where ***I*** is the identity matrix of size 4.

### Evolutionary dynamics

We have chosen a discrete imitation dynamic in a population of *N* individuals^45^,^46^. According to this dynamics, two individuals are selected at random from the population. The probability that the first individual adopts the strategy of the second one is given by a Fermi imitation probability function 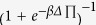
47,48. The parameter *β* represents the intensity of selection, *i.e*. the strength individuals base their decision to imitate the others, and ΔΠ is the difference of payoffs between both individuals. We have chosen *β* = 0.1 for all the calculations showed here. Note that the payoff is a measure of the success of individuals and therefore the higher the payoff the higher the probability of being imitated by others^18^,^19^.

A discrete dynamics like the one we are considering here always leads to an asymptotically homogeneous population. Since only mutations (invasions) can introduce new strategies, a homogeneous population is always an absorbing state. We calculate the probabilities of the different invasions as fixation probabilities, *i.e*. the probability that a single invader will eventually be imitated by all the rest of individuals, who play the resident strategy, and this under the assumption of the small mutation limit^49^. Note that due to its complexity we do not consider the possibility of mixed equilibria, like in other previous works^50^. This fixation probability is given by^19^,^51^


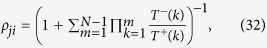


where *T*^+^(*k*) is the probability that an individual of the resident strategy *i* imitates a mutant one *j* and *T*^−^(*k*) is the probability that an individual of the mutant strategy imitates a resident one in a population of *k* individuals playing the resident strategy. These probabilities are obtained from the imitation probability defined previously:


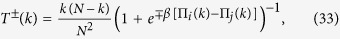


where Π_*i*_(*k*) and Π_*j*_(*k*) denote the average payoffs of the focal player and her opponent:


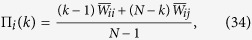



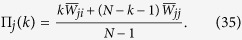


The probabilities defined by equation (32) determine a transition matrix of a Markov chain among strategies, assuming a sufficiently low mutation rate^49^. The normalized eigenvector associated with the eigenvalue 1 of that matrix provides the stationary distribution of strategies^46^,^52^, that represents the relative time the population spends adopting each of the strategies.

### Dominant strategies

One strategy *A* is risk-dominant against another one *B*^22^,^53^,^54^ when *π*_*A*,*A*_ + *π*_*A*,*B*_ > *π*_*B*,*B*_ + *π*_*B*,*A*_, where *π*_*i*,*j*_ is the payoff that an individual playing the *i*-strategy obtains when playing against another individual that plays the *j*-strategy. When the apology-forgiveness mechanism is introduced, these payoffs for the cooperating proposer (PC), defecting proposer (PD), and defecting acceptor (AD), in the absence of noise, are, respectively: *π*_*PC*,*PC*_ = −*ε*/2 + *b* − *c*, *π*_*PD*,*PD*_ = −*ε*/2, *π*_*AD*,*AD*_ = 0, *π*_*PC*,*PD*_ = −*ε*/2 + *γ* − *c*, *π*_*PD*,*PC*_ = −*ε*/2 + *γ* + *b*, *π*_*PC*,*AD*_ = −*ε* + *γ* − *c*, and *π*_*AD*,*PC*_ = −*γ* + *b*. Then the cooperative proposer is risk-dominant against the defective proposer when *γ* > *c* and against the defective acceptor when *γ* > *c* + 3*ε*/4 in the absence of noise and if all of them apologise when making a mistake.

## Additional Information

**How to cite this article**: Martinez-Vaquero, L. A. *et al*. Apology and forgiveness evolve to resolve failures in cooperative agreements. *Sci. Rep*. **5**, 10639; doi: 10.1038/srep10639 (2015).

## Supplementary Material

Supplementary Information
