# Supplementary material for: Apology and forgiveness evolve to resolve failures in cooperative agreements
Source: Sci Rep. 2015 Jun 9;5:10639. doi: 10.1038/srep10639 (PMC4460819; doi:10.1038/srep10639)
Supplement: Supplementary Information [file srep10639-s1.pdf]

Supplementary Information.
Apology and forgiveness evolve to resolve failures in cooperative agreements.

Luis A. Martinez-Vaquero[α,β], The Anh Han[γ],
Luís Moniz Pereira[λ] and Tom Lenaerts[α,β,*]

[α] AI lab, Computer Science Department, Vrije Universiteit Brussel, Pleinlaan 2, 1050 Brussels, Belgium
[β] MLG, Département d'Informatique, Université Libre de Bruxelles, Boulevard du Triomphe CP212, 1050 Brussels, Belgium
[γ] School of Computing, Teesside University, Borough Road, Middlesbrough, UK TS1 3BA
[λ] NOVA Laboratory for Computer Science and Informatics, Departamento de Informática, Faculdade de Ciências e Tecnologia, Universidade Nova de Lisboa, 2829-516 Caparica, Portugal

*corresponding authors: Tom.Lenaerts@ulb.ac.be

1 Commitments without apology-forgiveness mechanism

1.1 Stationary distribution of all strategies

In order to complement the Figure 1A of the Results section, we show in Supplementary Table 1 the frequencies corresponding to every strategy for the four scenarios separately for the usual values of parameters and noise 0.1 and 0.001. As one can see, strategies (P,C,AllD), (P,C,TFT) and (NC,-,AllD) are usually the main ones.

1.2 Influence of the different parameters of the model

In Results we showed the stationary distributions of the main strategies as a function of the noise for the four different scenarios. In Supplementary Figures 1–5 we complement that information showing the stationary distributions of strategies as a function of noise, b/c, ϵ, and δ for different parameters; Supplementary Figure 2 is focused on the case where commitments are not allowed. We see that an increase in the benefit-to-cost ratio leads to a decrease of the presence of pure defectors and an increase or maintenance of the level of proposers as well as the cooperation level. In PP and NP scenarios, proposers that play TFT outside the commitments benefit from this increase even more than those that defect in that situation, at least for low noise. Obviously the cheaper it becomes to set up a commitment the more successful the proposers are and vice versa for acceptors. Very low δ benefits those strategies that accept a commitment yet defect when playing the game, whereas if this value is increased, proposers that cooperate in commitments gain importance.

2 Commitments in the presence of apology-forgiveness mechanism

2.1 Costless apology

We started by analysing the effect of costless apology, which is equivalent to consider that players always apologise at no cost ($\gamma = 0$ and $q_{apo} = 1$). If we incorporate strategies that forgive in commitments with a given probability $q_{for} = q$, we see in Supplementary Figures 6 and 7 that the costless apology does not change substantially the conditions under which proposers are better than pure defectors. Forgivers do better when the benefit is high enough ($b \gtrsim 3$ for the PP scenario). In that situation, the higher the probability of forgiving the better at least for $q \lesssim 0.7$. We see the opposite behaviour for lower values of the benefit: forgiving is worse and worse when its probability is increased.

2.2 Apology-forgiveness

In order to complement the Figure 3 of the Results, we show in Supplementary Figure 8 the stationary distributions of the main strategies under the presence of apology-mechanism for the four scenarios. We confirm what we discussed in Results and Conclusions: the apology-forgiveness mechanism only works under sincere apologies for all the scenarios.

	$\epsilon = 10^{-3}$				$\epsilon = 10^{-1}$			
	**NN**	**PN**	**NP**	**PP**	**NN**	**PN**	**NP**	**PP**
(P,C,AllC)	**0.36**	0.04	0.03	0.02	**0.11**	<0.01	0.01	<0.01
**(P,C,AllD)**		**0.22**	**0.25**	**0.28**		**0.11**	**0.27**	**0.38**
(P,C,ATFT)		0.06	0.06	0.04		0.03	0.02	0.01
**(P,C,TFT)**		**0.14**	**0.19**	**0.16**		**0.04**	**0.14**	**0.11**
(P,D,AllC)	<0.01	<0.01	<0.01	<0.01	<0.01	<0.01	<0.01	<0.01
(P,D,AllD)		<0.01	0.04	0.04		0.01	0.03	0.04
(P,D,ATFT)		<0.01	<0.01	<0.01		<0.01	<0.01	<0.01
(P,D,TFT)		<0.01	0.02	0.02		<0.01	0.01	<0.01
(A,C,AllC)	0.01	0.01	0.01	0.01	0.01	0,02	<0.01	<0.01
(A,C,AllD)	0.06	0.02	0.02	0.02	0.20	0.12	0.15	0.12
(A,C,ATFT)	0.01	<0.01	<0.01	<0.01	0.19	0.02	0.01	<0.01
(A,C,TFT)	0.15	0.12	0.12	0.12	0.09	0.09	0.06	0.06
(A,D,AllC)	<0.01	<0.01	<0.01	<0.01	<0.01	<0.01	<0.01	<0.01
(A,D,AllD)	0.07	0.03	0.07	0.06	0.17	0.08	0.1	0.07
(A,D,ATFT)	<0.01	<0.01	<0.01	<0.01	0.01	0.01	<0.01	<0.01
(A,D,TFT)	0.11	0.08	0.04	0.05	0.07	0.06	0.02	0.02
(NC,-,AllC)	<0.01	<0.01	<0.01	<0.01	<0.01	<0.01	0.01	<0.01
**(NC,-,AllD)**	**0.08**	**0.13**	**0.03**	**0.08**	**0.20**	**0.26**	**0.06**	**0.11**
(NC,-,ATFT)	<0.01	<0.01	<0.01	<0.01	0.02	0.02	0.02	<0.01
(NC,-,TFT)	0.12	0.09	0.09	0.07	0.08	0.07	0.05	0.03

Supplementary Table 1: Stationary distribution of all the strategies for all the scenarios separately. The strategies plots in Figure 1a of the Results section are marked in bold. We assumed $\omega = 0.9$, $b/c = 2$, $\epsilon = 0.25$, and $\delta = 4$.

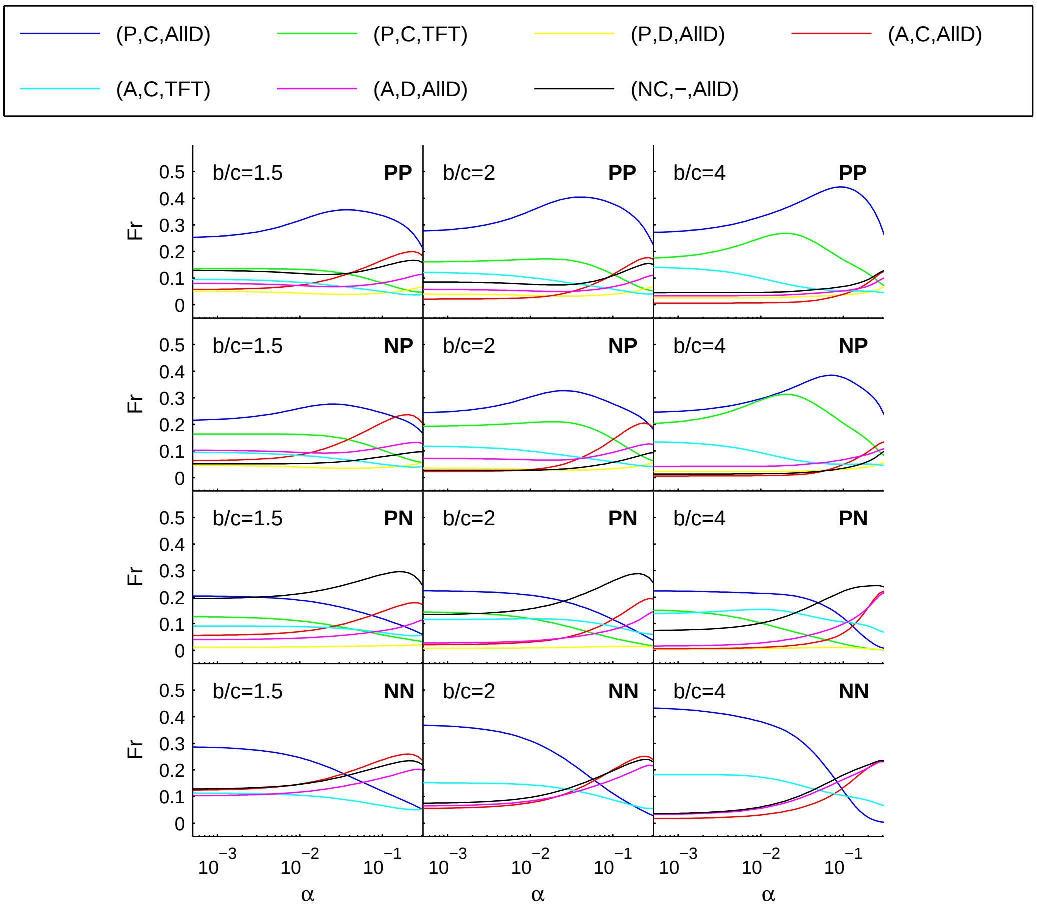

Supplementary Figure 1: Stationary distribution of the main strategies as a function of the noise. We consider different benefit-to-cost ratio and scenarios, and assume $\omega = 0.9$, $\epsilon = 0.25$, and $\delta = 4$.

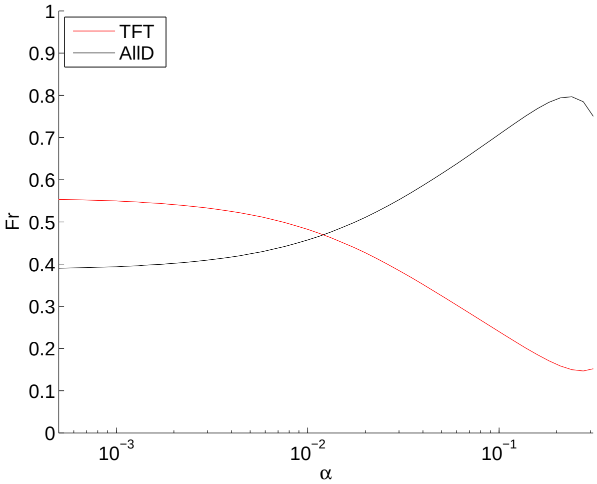

Supplementary Figure 2: Stationary distribution of TFT and AllD as a function of the noise when commitments are not allowed. We assumed $b/c = 2$ and $\omega = 0.9$.

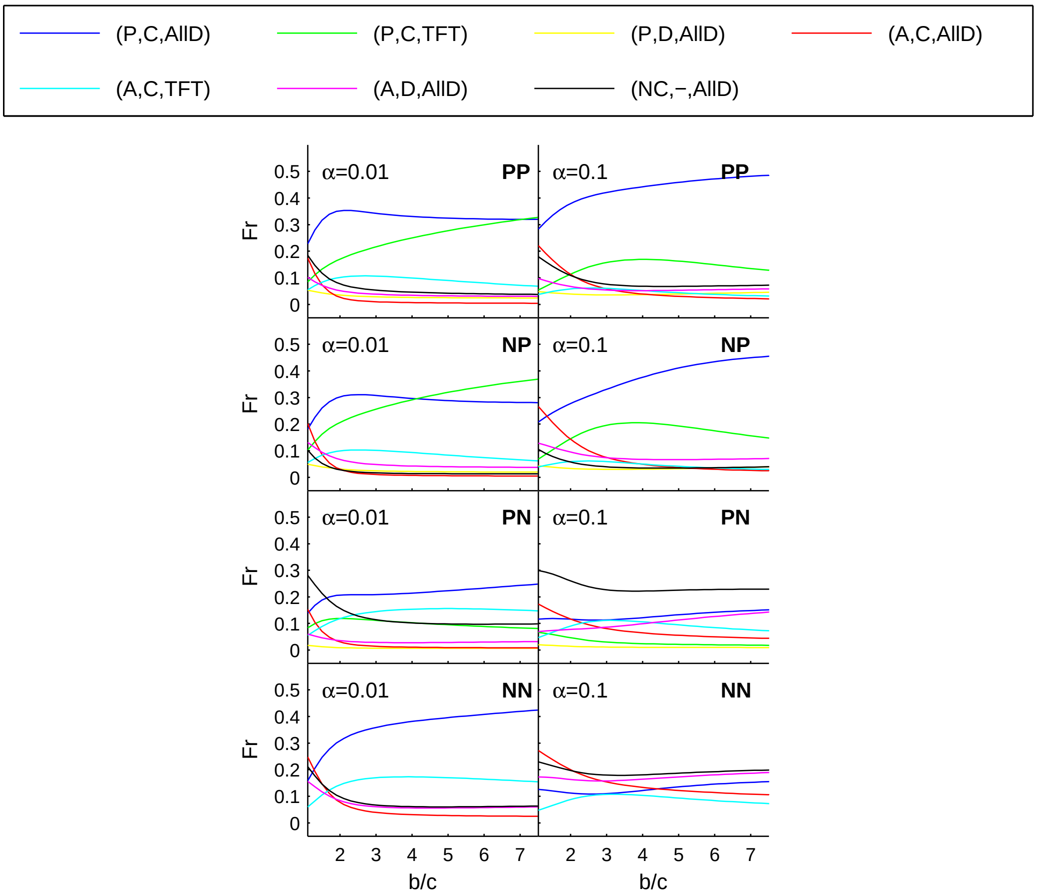

Supplementary Figure 3: Stationary distribution of the main strategies as a function of the benefit-to-cost ratio. We consider different noise and scenarios, and assume $\omega = 0.9$, $\epsilon = 0.25$, and $\delta = 4$.

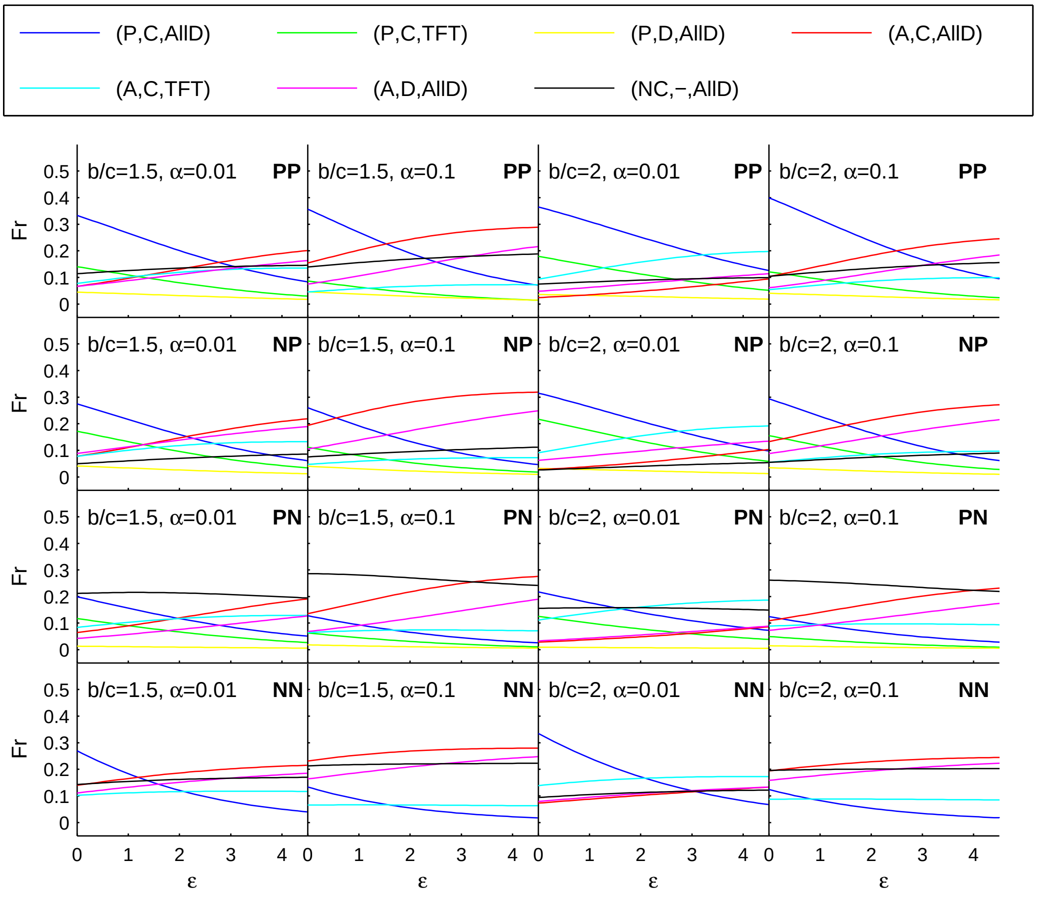

Supplementary Figure 4: Stationary distribution of the main strategies as a function of ϵ. We consider different noise, benefit-to-cost ratio, and scenarios, and assume $\omega = 0.9$ and $\delta = 4$.

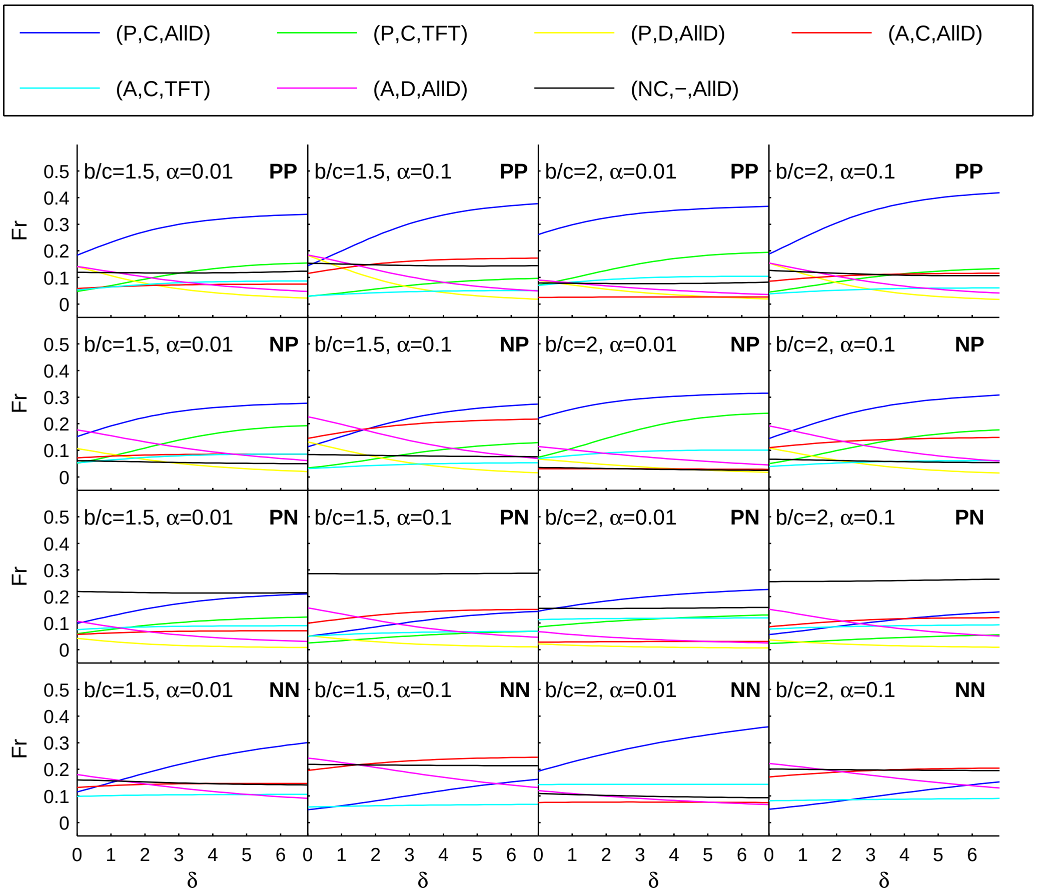

Supplementary Figure 5: Stationary distribution of the main strategies as a function of δ. We consider different noise, benefit-to-cost ratio, and scenarios, and assume $\omega = 0.9$ and $\epsilon = 0.25$.

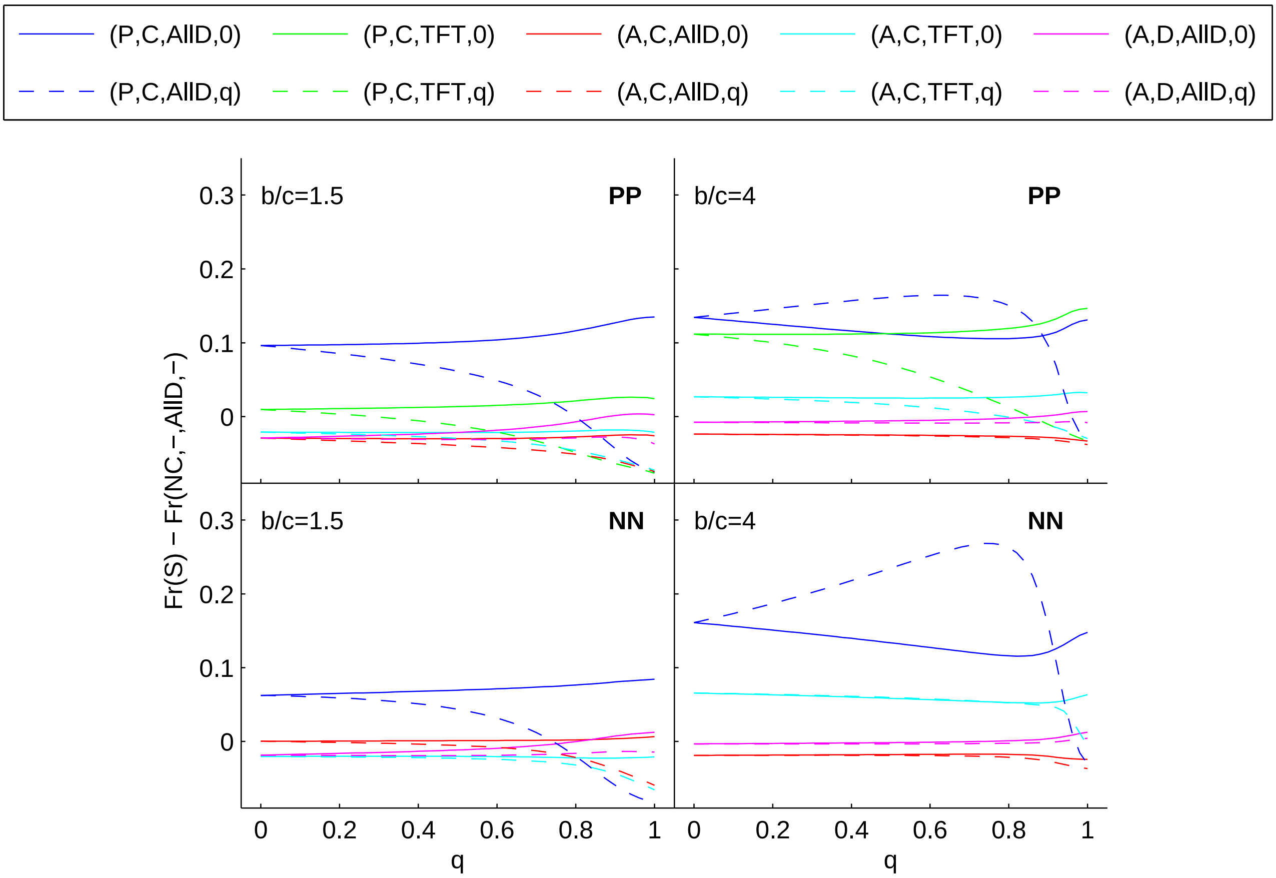

Supplementary Figure 6: Stationary distribution of the main strategies as a function of the probability of forgiveness q. We consider different benefit-to-cost ratio in the presence of costless apology for PP and NN scenarios.

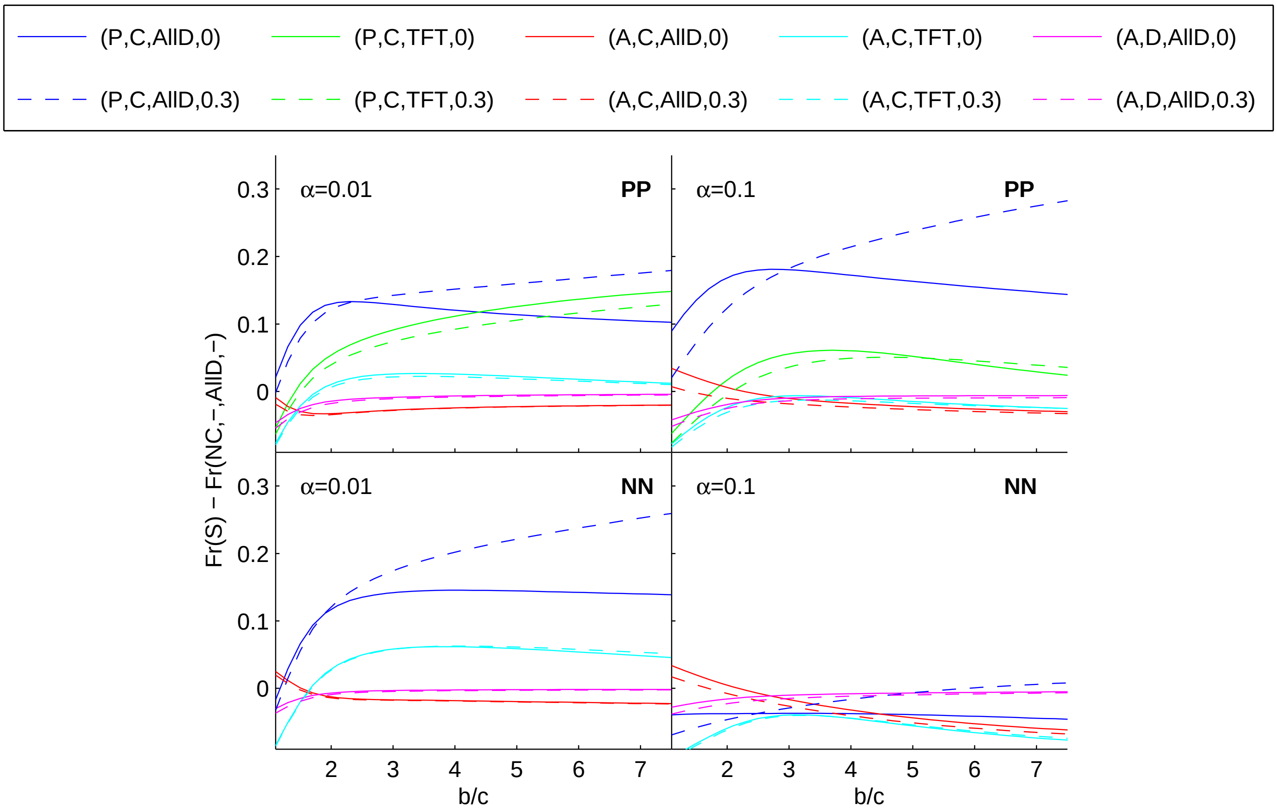

Supplementary Figure 7: Stationary distribution of the main strategies as a function of the benefit-to-cost ratio. We consider different noise in the presence of costless apology for PP and NN scenarios.

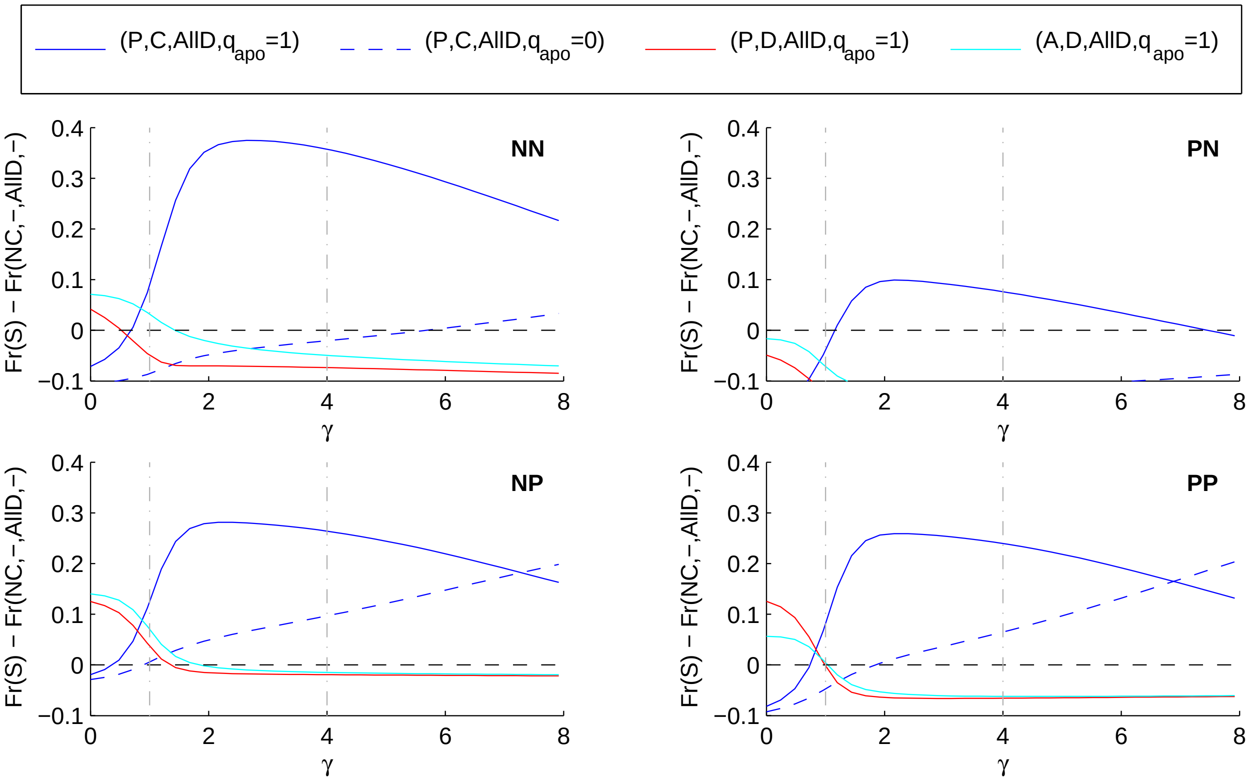

Supplementary Figure 8: Success of forgiveness for different apologies in different scenarios. Stationary distribution of the main strategies with respect to the stationary distribution of the pure defectors as a function of the apology cost for the different scenarios. Vertical dashed lines mark the values of c and δ. We assumed $\omega = 0.9$, $b/c = 2$, $\alpha = 0.1$, $\epsilon = 0.25$, and $\delta = 4$.

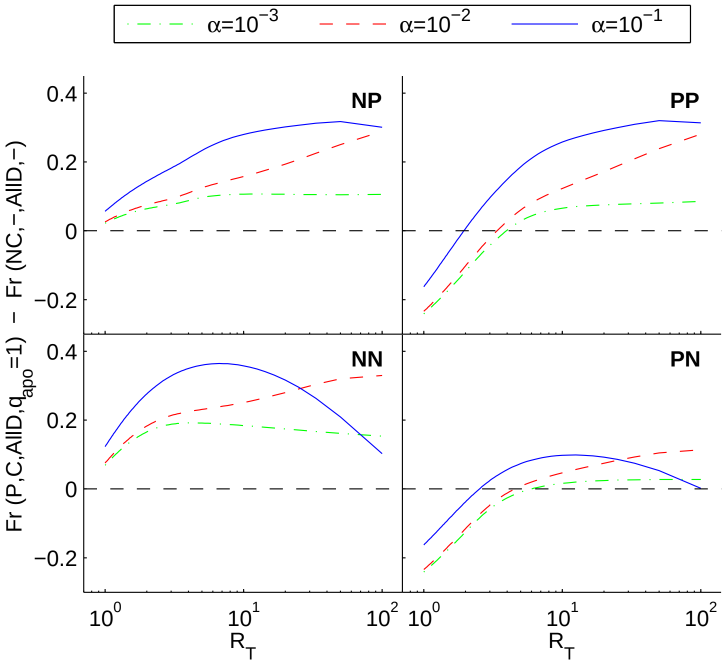

Supplementary Figure 9: Influence of the number of rounds in the success of forgiveness. Stationary distribution of the $(P, C, AllD, q_{apo} = 1)$ strategies with respect to the stationary distribution of the pure defectors as a function of the total number of rounds for the four scenarios. We assumed $b/c = 2$, $\epsilon = 0.25$, $\delta = 4$, and $\gamma = 2$.

In Supplementary Figures 9-11 we examined how the frequency of $(P, C, AllD, q_{apo} = 1)$ changes as the average number of rounds in the IPD. We can see in the NP and PP scenarios, that, when individuals can play after the commitment is broken, this frequency generally increases with R_T. When the players cannot play after the commitment is broken, *i.e.* in NN and PN scenarios, a similar observation is only seen for sufficiently low levels of noise. When noise is large, the frequency of these apologising commitment proposers drops when R_T reaches certain threshold but it remains higher than when $R_T = 1$, *i.e.* for the one-shot PD, for a wider range of R_T. That said, arranging commitments in long-term interactions is more beneficial than in the one-shot one, especially when one takes apology during and revenge after the commitment is broken into account. One of the reasons that this occurs is because the cost of arranging commitment ϵ is paid only once at the beginning of the IPD, thereby reducing its detrimental (per round) impact on a proposer for increasing R_T. This cost, if becoming too large, is highly detrimental for cooperation in the one-shot PD. This interesting observation becomes even clearer when we look at the results for varying ϵ.

We also checked the influence of β repeating the same analysis as in Figure 3 of the Results but for $\beta = 0.001$ and $\beta = 1$ in Supplementary Figures 12 and 13, respectively. One can observe that β has no important impact on the conclusions we obtain for our study of the apology-forgiveness mechanism.

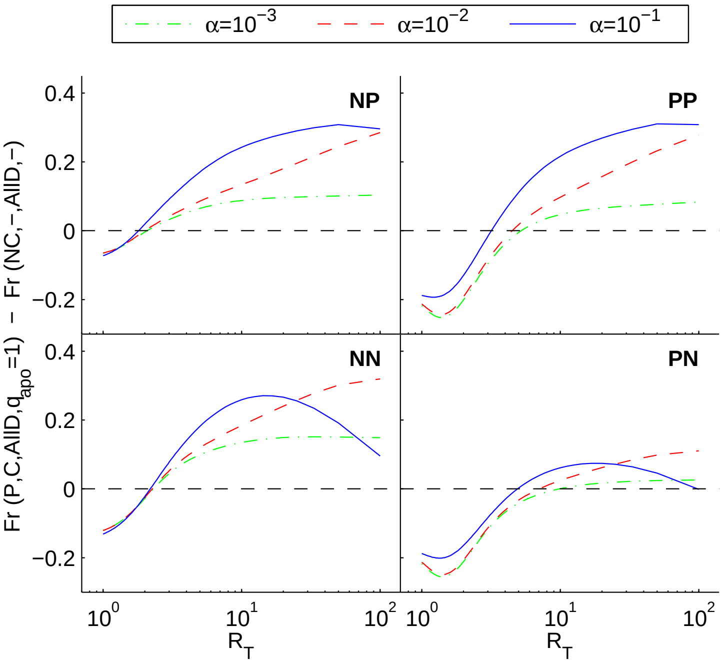

Supplementary Figure 10: Influence of the number of rounds in the success of forgiveness for $\epsilon = 1$. Same as Supplementary Figure 9 but for $\epsilon = 1$.

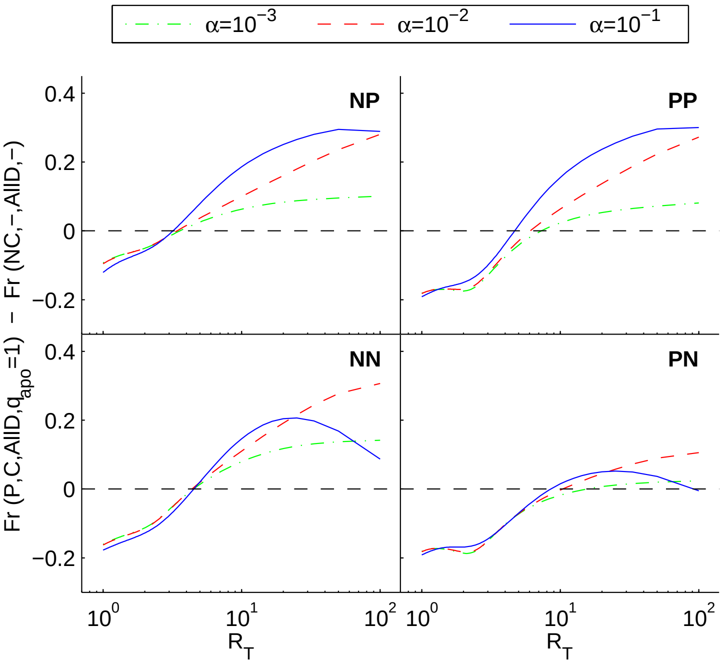

Supplementary Figure 11: Influence of the number of rounds in the success of forgiveness for $\epsilon = 2$. Same as Supplementary Figure 9 but for $\epsilon = 2$.

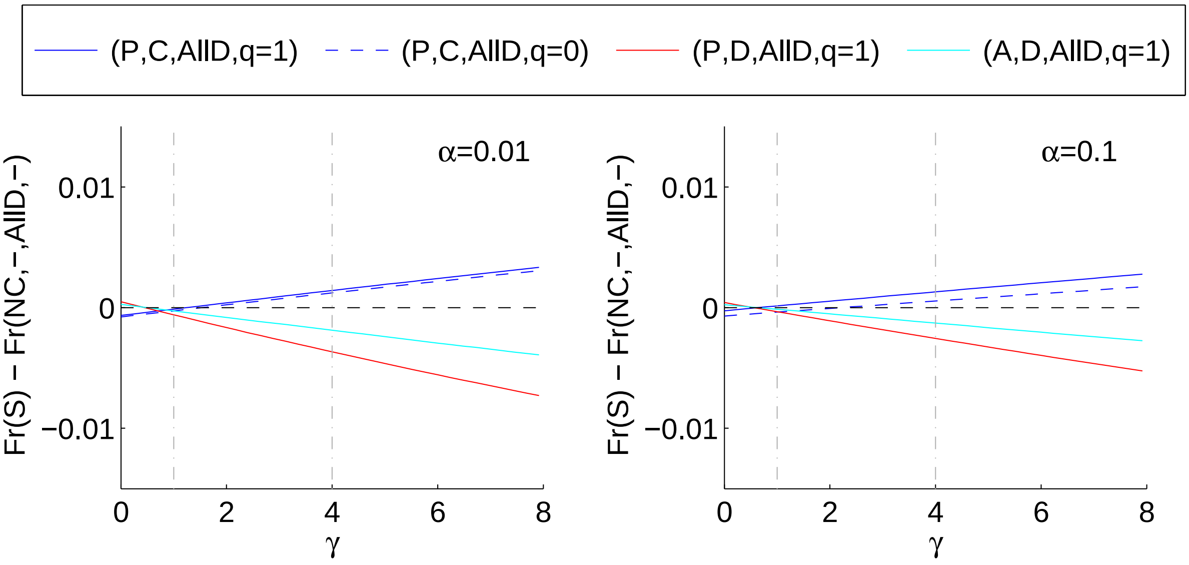

Supplementary Figure 12: Same as Figure 3 in the manuscript but for $\beta = 10^{-3}$.

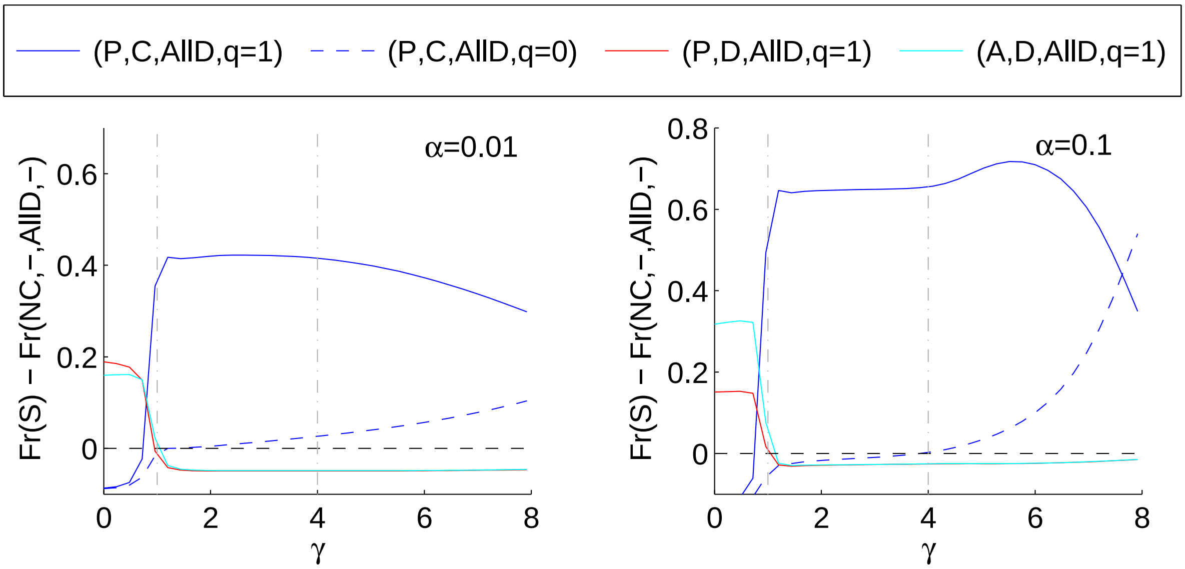

Supplementary Figure 13: Same as Figure 3 in the manuscript but for $\beta = 1$.
